# Interventions to promote exclusive breastfeeding among young mothers: a systematic review and meta-analysis

**DOI:** 10.1186/s13006-020-00340-6

**Published:** 2020-12-01

**Authors:** Christa Buckland, Debra Hector, Gregory S. Kolt, Paul Fahey, Amit Arora

**Affiliations:** 1 School of Health Sciences, Western Sydney University, Locked Bag 1797, Penrith, NSW 2751 Australia; 2 Cancer Australia, Surry Hills, NSW 2010 Australia; 3 Translational Health Research Institute, Western Sydney University, Locked Bag 1797, Penrith, NSW 2751 Australia; 4 Discipline of Child and Adolescent Health, Sydney Medical School, Faculty of Medicine and Health, The University of Sydney, Westmead, NSW 2145 Australia; 5 Oral Health Services, Sydney Local Health District and Sydney Dental Hospital, NSW Health, Surry Hills, NSW 2010 Australia

**Keywords:** Exclusive breastfeeding, Interventions, Young mothers, High income countries, Systematic review

## Abstract

**Background:**

Exclusive breastfeeding rates in many high-income countries are considerably lower than the World Health Organization recommendations. Younger mothers are less likely than older mothers to exclusively breastfeed or to exclusively breastfeed for a long duration. This systematic review explores interventions to increase the rate of exclusive breastfeeding among young mothers in high-income countries.

**Methods:**

A systematic search of the following databases was completed in August 2020: CINAHL, PubMed, MEDLINE, ProQuest, PsychInfo, Web of Science, Cochrane, Scopus and Embase. A manual search of the reference lists of all the included studies and published systematic reviews was also performed. The Cochrane Collaboration Risk of Bias Tool was used to assess the quality of the included studies. A random effects model meta-analyses was applied. Heterogeneity of outcomes between the studies was assessed using both the χ^2^ test and the I^2^ statistic.

**Results:**

Of 955 records identified in the search, 392 duplicates were removed, and nine studies met the inclusion criteria. Seven studies were randomised controlled trial (RCTs) and two were quasi-experimental in design. Eight were conducted in the United States. The interventions included peer counselling, telephone support, massage, gift packs, financial incentive and antenatal education. Most studies included a combination of strategies, peer counselling being the most common. A meta-analysis of four of nine included studies did not detect a difference in rate of exclusive breastfeeding to 3 months postpartum (RR 1.44; 95% CI 0.82, 2.55; *p* = 0.204).

This review is limited by the relatively few studies which met the inclusion criteria and the small sample sizes of most included studies. High rates of attrition and formula supplementation among the participants made it difficult to detect a statistically significant effect. Consistency in follow up times would enable more studies to be included in a meta-analysis.

**Conclusions:**

Peer counselling was the most promising strategy associated with higher rates of exclusive breastfeeding. However, further studies are needed to understand the breastfeeding experiences of young mothers. Young mothers should be targeted specifically in intervention studies.

**Supplementary Information:**

The online version contains supplementary material available at 10.1186/s13006-020-00340-6.

## Background

Increasing the rates of breastfeeding and in particular, exclusive breastfeeding (EBF) to 6 months, is a public health priority across the world [1]. The World Health Organization (WHO) defines ‘exclusive breastfeeding’ as an infant receiving only breast milk (whether that be directly from the breast, from a bottle or from a donor/wet nurse) [2]. This definition allows the infant to receive prescribed drops or syrups (vitamins, minerals, medicines) but nothing else [2]. The WHO recommends that infants be exclusively breastfed until 6 months of age and continue to be breastfed, in conjunction with solids, for up to 2 years and beyond if desired [3].

Although any amount of breastfeeding provides multiple health benefits for both the mother and infant [1, 4–11], EBF provides greater benefits than partial breastfeeding during the first 6 months of life, particularly in relation to preventing gastrointenstinal and respiratory infections [5, 12]. Kramer and Kakuma [5] conducted a systematic review of the literature and concluded that 6 months was the optimal duration for EBF and had significantly more health benefits than EBF to 3 or 4 months. Further, there is evidence to show that the longer the duration of EBF (up to 6 months), the greater the health benefits it may provide [13]. It is also established that supplementation affects the mother’s milk supply and thus EBF is associated with longer duration of any breastfeeding [14].

Although the benefits of EBF are well-established, globally only 40% of infants under the age of 6 months are exclusively breastfed [15]. In most high-income countries the proportion of babies exclusively breastfed may be significantly less than the global average [4]. The Centers for Disease Control and Prevention reported that 24.9% of babies in the US in 2018 were exclusively breastfed to 6 months [16]. In Australia, the *National Infant Feeding Survey* for 2010 reported only 15.4% of babies were exclusively breastfed for five completed months (to 5 months) and only 2.1% were exclusively breastfed for the recommended six completed months (to 6 months), despite high breastfeeding initiation rates of 90% [17]. Low rates of EBF to 6 months are reported in other high-income countries such as the United Kingdom (1%), Norway (7%), Denmark (17%) and the Netherlands (17%) [18].

Younger age has been found to be associated with poorer breastfeeding practices in a large number of studies. For example, Jones et al. [19] found that mothers in the US aged 30 years or older were more than twice as likely, compared with mothers 20 years or younger, to exclusively breastfeed to 6 months (18.0% vs 8.3%). Similarly, the *2010 Australian National Infant Feeding Survey* reported that the proportion of mothers aged 24 years or younger who exclusively breastfed to 5 months (6.2%) was less than one-third that of mothers aged 35 years or older (19.2%) [17]. The *Infant Feeding Survey, UK – 2010* reported that mothers aged 24 years or younger were less likely to exclusively breastfeed at each month of age to 6 months [20].

A broad range of interventions and programs has been implemented in various contexts to promote breastfeeding initiation, duration, and exclusivity, with varying degrees of success. Interventions have included strategies such as peer counselling, professional counselling, online support, phone support, antenatal breastfeeding education, multimedia approaches, motivational interviewing, breastfeeding-friendly hospital practices, breastfeeding-friendly workplaces, and parental leave policies [21–28]. Despite this extant research, young mothers do not appear to be well represented in these intervention studies. Most interventions do not target young mothers specifically and some even exclude an important sub-group of young mothers (adolescent mothers aged less than 18 years). However, given that young mothers exhibit particularly low rates of EBF in high-income countries [17, 19, 20, 29], they are an important population on which to focus.

The purpose of this systematic review and meta-analysis is to examine the range and effectiveness of interventions which have been designed to increase rates of EBF among young mothers in high-income countries. The specific focus on high-income countries allows the findings to directly inform the development and implementation of an intervention in a high-income country setting.

## Methods

This review was conducted based on the Preferred Reporting Items for Systematic Reviews and Meta-analysis (PRISMA) guidelines (see Additional file 1 for complete PRISMA checklist) [30]. The protocol for this systematic review is registered with PROSPERO International Prospective Register of Systematic Reviews (2018: CRD42018083989) [31]. The Population Intervention Comparator Outcome Study Design (PICOS) criteria were used to devise the review question and search terms [32]. The PICOS table is presented in Table 1. A combination of MeSH terms and keywords was drafted and peer reviewed for comprehensiveness. The search strategy was pre-tested in the MEDLINE database (see Additional file 2) and subsequently adapted to the syntax and subject headings of all other databases.

**Table 1:** Population Intervention Comparator Outcome Study Design (PICOS) table

**P – Population**	Young mothers (mothers aged 24 years or less) in high-income countries; infants 0–6 months
**I – Intervention**	Any intervention
**C – Comparator**	Any comparator (most commonly, Usual Care)
**O – Outcome**	Increasing exclusive breastfeeding rates
**S – Study Design**	Randomized Controlled Trials and Quasi Experimental designs (prospective with a control group)

### Information sources

A systematic search of the following databases was conducted: MEDLINE (OVID), Scopus, Web of Science (ISI), PubMed, PsychInfo, ProQuest Central, Cochrane Central Register of Controlled Trials (CENTRAL) (The Cochrane Library), Embase (OVID), and Cumulative Index to Nursing and Allied Health Literature (CINAHL) (EBSCO). Keywords used in the searches included: exclusive; breastfeeding; infant feeding; adolescents; young mothers and randomized controlled trial. Appropriate truncations and search functions (such as subject headings) were utilized and modified according to the database. Date limitations were not applied to the search. The primary search was conducted in February 2018 and the final search was completed in August 2020 to ensure that more recent studies would not be omitted. Additionally, a manual search of the reference lists of all the included studies and published systematic reviews was performed.

### Eligibility criteria

Inclusion criteria were based on PICOS criteria (see Table 1; Additional file 3). Studies were included if they were conducted in a high-income country, were an RCT or had a quasi-experimental design, were published in a peer reviewed journal, were written in the English language, measured EBF and had a sample population of mothers with a mean or median age of less than 25 years. The mean or median age of the sample population was used to identify studies which had a large proportion of young mothers (i.e., younger than 25 years). If a mean or median age was not reported, the study was excluded. All studies which met the criteria except for age were reviewed for sub-group analysis by age. Studies with follow-up times of up to 6 months were included. The introduction of solids is recommended around 6 months of age and therefore EBF is rare and usually unnecessary after this time [3]. Quasi-experimental designs were deemed acceptable if they were prospective and had a control group, as randomization is not always possible or appropriate in breastfeeding research. The World Bank classification of countries was used to identify high-income countries [33]. No restriction on publication dates was applied as the review aimed to assess all published studies related to the aims.

### Study selection

**Fig. 1:**
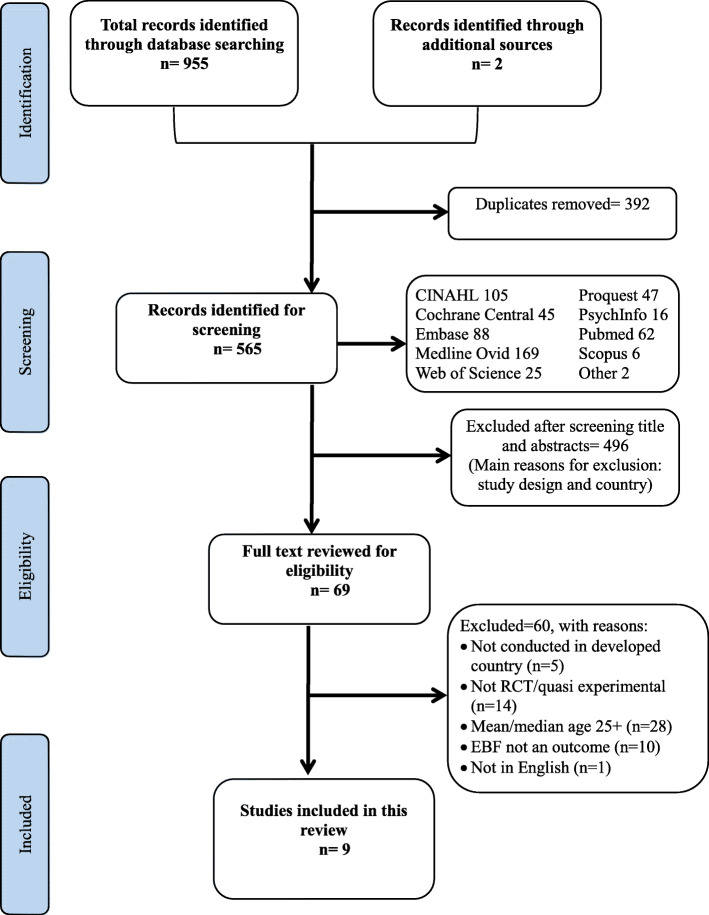
PRISMA flowchart of study selection process

Studies identified through the electronic databases were exported to Endnote X8 for removing duplicates, screening, and selection [34]. Two reviewers (CB and AA) independently and in duplicate screened the studies against the inclusion criteria mentioned above. Full texts of the articles that met the inclusion criteria were independently assessed by two reviewers (CB and AA). In case of uncertainty regarding the eligibility and study selection, the study authors were contacted to seek additional information. A total of three contact attempts were made, and if no response was received, the articles were screened for eligibility based on the information available. Any disagreements were resolved through discussion with two further reviewers (GSK and DH). The reasons for excluding studies that did not meet the inclusion criteria were recorded (Additional file 4). The search strategy resulted in nine studies being included in this review. This process is summarized in the PRISMA flow diagram (Fig. 1).

### Data collection process and data items

A standardized data extraction form was developed and pilot-tested independently by two reviewers (CB and AA). Extracted data included first author, publication year, country, study design, sample size, sample characteristics, intervention description, comparison, and reported outcomes. Data extraction was conducted primarily by two reviewers (CB and AA) independently. GSK and DH provided feedback and resolution for any disagreements. In case of missing data and/or uncertainties, the study authors were contacted for further information with a maximum of three attempts. The extracted data are listed in Table 2.

**Table 2:** Summary of included studies targeting young mothers in high-income countries to increase EBF

Study, Country	Design	n	Sample characteristics	Intervention Description	Comparison	Reported Outcomes
Arlotti et al. (1998), USA [35]	Quasi experimental	36 Exp: 18 Con: 18	Convenience sample: Prenatal and postpartum mothers who were enrolled in Women, Infants and Children (WIC) program. Low income population in North Florida. Mean age: 23.3 (SD 4.4) Age range: 15–36	Support from peer counsellors a few days after delivery, then at 2 weeks, 1 month, 2 months and 3 months postpartum via telephone, letter or in person at WIC office.	No counsellor/Usual care at same time intervals.	Mean rates of EBF^a^, experimental VS control: • at 2 weeks, 53% vs 17% • at 1 month, 40% vs 27% • at 2 months, 33% vs 13% • at 3 months, 17% vs 6%
Bunik et al. (2010), USA [36]	RCT	341 Exp: 161 Con: 180	Convenience sample of medically underserved (88% Hispanic/Latino) mothers, recruited from a subsidized hospital in Denver, Colorado. Median age: 22 Age range: 18+ (range not provided)	Two weeks of daily telephone calls by trained bilingual nurses using a culturally informed script. Outcomes assessed by maternal report at 1, 3 and 6 months postpartum.	Usual care	No mothers EBF. There was no significant difference in any BF^b^ or predominant^c^ BF between the groups. Participants in the experimental group, who planned to EBF were more likely to “predominantly” BF at 1, 3 and 6 months. This trend was not seen in the control group.
Chapman et al. (2013), USA [37]	RCT	206 Exp: 103 Con: 103	206 pregnant, overweight/obese, low-income women were recruited. 154 met inclusion criteria at delivery. Pre-pregnancy BMI = 27.0+ Mostly Hispanic, singleton pregnancy, unemployed, high school education, vaginal birth. Median age (exp): 23 (IQR 21–28) Median age (con): 25 (IQR 22–31) Age range: not reported	Specialized breastfeeding peer counselling (SBFPC) 3 x Prenatal, daily in hospital after delivery and up to 11 x postnatal sessions plus routine care.	Brief breastfeeding discussions during routine prenatal visits at the clinic	Experimental group had higher rates of EBF at some time points but the difference was not significant. At 2 weeks experimental group had higher rates of any BF (93% vs 84%, *P* = 0.09) and ≥ 50% of feeds as breast milk (81% vs 67%, *P* = 0.08).
Di Meglio et al. (2010), USA [38]	RCT	78 Exp: 38 Con: 40	Breastfeeding mothers with a healthy full-term infant recruited from hospitals in Rochester, New York Mean age (exp): 18.4 (SD 1.3) Mean age (con):18.2 (SD 1.4) Age range: Not reported but participants were < 20 years old.	Telephone support at 2, 4, and 7 days and 2, 3, 4, and 5 weeks post discharge from the hospital; content based on WIC’s breastfeeding promotion efforts, gave telephone numbers. Facilitators: Adolescent peer counsellors trained by La Leche League	Usual care	There was no significant difference for any BF duration between the groups. 68% of experimental group and 75% of control group received supplements at the hospital prior to discharge. Out of the remaining participants, duration of EBF was significantly increased in the experimental group (median 35 days vs 10 days, *P* < 0.01).
Pugh et al. (2002), USA [39]	RCT	41 Exp: 21 Con: 20	Low-income mothers receiving financial medical assistance support, recruited from hospitals in mid-Atlantic region. Predominantly African American and mostly single. Mean age (exp): 20.86 (SD 3.58) Mean age (con): 22.35 (SD 4.98) Age range: not reported	Usual care plus supplementary visits from a community health nurse/peer counsellor team, daily during hospitalization and then at home during weeks 1, 2, and 4; peer counsellors provided support over the phone twice weekly through week 8 and weekly through month 6. Facilitators: Community health nurse and peer counsellor	Usual care included support from hospital nurses, telephone “warm line” and one visit by a lactation consultant if they birth on a weekday.	More mothers in the experimental group EBF their infants however it wasn’t significant due to the small sample size. • 45% (*n* = 9) vs 25% (*n* = 5) at 3 months • 30% (*n* = 6) vs 15% (*n* = 3) at 6 months X^2^ = 1.29–1.75; *P* = 0.09–0.12
Serano et al. (2010), Chile [40]	Quasi-experimental comparative panel design - RCT deemed not appropriate due to risk of contamination	100 Exp: 35 Con: 65	Mothers with healthy newborns recruited from 3 health clinics in Santiago, Chile. Low-income community. Predominantly unemployed. Mean age: 24.3 (SD 5.9) Mean age (con): 24.08 (SD 5) Age range: 14–43	During 2nd well-child clinic visit, nurses provided video instruction on how to massage baby. Mothers also received a booklet and were encouraged to massage their baby for 10–15 min once a day from Day 15.	No treatment	At age 2 months, massage group infants weighed significantly more than control-group infants. There were no weight differences between the 2 groups at age 4 months. There were no differences between the 2 groups on the incidence of exclusive maternal breast-feeding at age 2 or 4 months.
Snell et al. (1992), USA [41]	RCT	88 Exp: 50 Con: 38	Hispanic mothers recruited from Family Centered Perinatal Care Unit in California. Mean age (exp): 25.2 (18–43) Mean age (con): 24.3 (18–36) Age range: 18–43	The study period (12 weeks) was divided into 2-week blocks and randomly assigned to experimental (non-gift pack) and control (gift pack) groups. Mothers were then interviewed (by telephone) at 1 and 3 weeks old.	The control group received a gift pack including samples of formula.	• EBF at 1 week: 80% vs 68% • EBF at 3 weeks: 68% vs 33% Supplementing or bottle feeding: • At 1 week: 20% vs 32% • At 3 weeks: 32% vs 66%
Wambach et al. (2011), USA [42]	RCT	390 Exp: 128 Con1: 128 Con2: 134	Enrolled adolescent mothers in their second trimester from prenatal clinics and school settings. All first-time mothers. Predominantly African American, and single. Mean age: 17 (SD 0.9) Age range: 15–18	Co-delivered by lactation consultant (LC) and peer counsellor (PC). Two prenatal classes consisting of content from Breastfeeding Educated and Supportive Teen club (BEST) curriculum, in-hospital support and postpartum telephone calls at 4, 7, 11, 18 days and 4 weeks. Participants also received a free double electric pump.	Con1 = attention control delivered by nurse and PC, included 2 prenatal classes, telephone support, and in-hospital PC visit (not on the topic of BF). Con2 = usual care	• Median BF duration was significantly higher in experimental group: 177 (exp) vs 42 (con1) vs 61 (con2) days. *P* < 0.001 • No significant differences for EBF initiation or duration • High overall rates of supplementation. At 3 weeks postpartum 69% (exp), 70% (con1), 82% (con2) were supplementing with formula.
Washio et al. (2017), USA [43]	Randomized two-arm parallel group design	36 Exp: 18 Con: 18	Puerto Rican mothers enrolled in a WIC program, who initiated breastfeeding. Mean age (exp): 24.1 (SD 4.7) Mean age (con): 23.0 (SD 4.6) Age range: not reported	Standard breastfeeding services from WIC – onsite LC, bilingual PC, weekly peer support meetings, free breast pump, enhanced food package for BF mothers. Plus, monthly financial incentives (total = $270) if they could demonstrate BF or pumping.	Standard breastfeeding services from WIC	Significantly higher rates of BF in experimental group vs control: • 89% vs 44% at 1 month • 89% vs 17% at 3 months • 72% vs 0% at 6 months • Mean duration of BF was 149 (exp) vs 49 (con) days. (P < 0.001) No significant difference in self-reported EBF rate.

*Notes & Abbreviations:*

^a^*EBF* Exclusive breastfeeding

^b^*BF* Breastfeeding

^c^Predominant breastfeeding in this study was defined as feeding 4 oz. or less of formula per day

*Exp* Experimental/intervention group

*Con* Control group

*PC* Peer counsellor

*LC* Lactation consultant

*WIC* Women, Infants and Children Office

### Quality assessment

The Cochrane Collaboration Risk of Bias Tool (RoBT) was used to assess the quality of the included studies (Additional file 5) [44]. The RoBT is a systematic process whereby studies can be measured against specific criteria. Each study was given a rating of high, low or unclear in the following domains: selection bias, performance bias, detection bias, attrition bias, and reporting bias. The RoBT also allows the assessors to report any other biases which may not fall into these five domains.

The RoBT was completed by two reviewers (CB and AA) independently. If there were disagreements, consensus was reached through discussion with two other reviewers (GSK and DH). Study authors were contacted in the event of insufficient details being available to confidently assess the methodological quality; and if a response was not received after three attempts, the study quality was assessed based on the available information.

### Data synthesis

Due to the diverse range of interventions, the low number of included studies, and that many studies were mixed-mode interventions comprising several strategies, it was not possible to conduct a meta-analysis on a specific intervention. Thus, data were analyzed to compare the effectiveness in terms of EBF outcomes of ‘any intervention’ versus ‘no intervention’. The most commonly reported outcome measures were EBF rates to 1-, 3-, and 6-months after birth. The number of women EBF and the number of women not EBF were extracted at each of these timepoints. Papers not reporting any of these outcomes were excluded. The data extracted from the included articles and used in the meta-analyses are listed in Additional file 6.

The relative risk of EBF at each of the three timepoints was determined using random effects meta-analyses. Results were reported as forest plots displaying both the relative risk and 95% confidence interval for each individual study and the equivalent pooled results.

Heterogeneity of outcomes between the studies was assessed using both the χ^2^ test and the I^2^ statistic. *P*-values less than 0.05 from the χ^2^ test were interpreted as statistically significant evidence of heterogeneity. I^2^ statistics of 0–40% were considered ‘not important’, 30–60% ‘may represent moderate heterogeneity’, and 75–90% ‘considerable heterogeneity’ [45]. Sensitivity analyses were performed to exclude studies whose quality rating was ‘poor’ in order to evaluate the impact of study quality on the results. Neither sub-group analyses nor assessment of publication bias could be undertaken due to the low number of included studies (< 10) [46, 47]. Analyses were performed using the metafor package in R software [48].

## Results

A total of 955 titles and abstracts were identified across all selected electronic databases. Two additional titles and abstracts were identified through a manual search of the reference lists of systematic reviews found in the database search. After removal of duplicates (*n* = 392), a total of 565 titles and abstracts were identified for further examination. The most common reasons for exclusion at this stage were the study design and country in which the study was conducted. Sixty-nine studies were identified as meeting the inclusion criteria for full text reading, and of these, nine studies were included in the systematic review and four in the meta-analyses. The 60 excluded studies and the reason for exclusion are presented in Additional file 4. All studies which met the criteria except for age were reviewed for sub-group analysis by age but none of them reported on this (possibly due to small numbers of younger mothers) and hence remained excluded. The inter-reader agreement for the entire search process was 100%. A PRISMA flow diagram was constructed showing the identification, screening, eligibility and included studies (Fig. 1).

### Characteristics of included studies

Nine studies were included in this review [35–43]. Of these, eight studies were conducted in the USA [35–39, 41–43] and one in Chile [40]. Seven studies were RCTs [36–39, 41–43] and two were of a quasi-experimental study design [35, 40]. The publication dates ranged between 1992 and 2017. The studies had relatively small sample size, ranging from 36 to 390 participants. Six of the studies had 100 or fewer participants [35, 38–41, 43]. The follow up time ranged from 3 weeks to 6 months. The mean or median age of mothers ranged from 17 to 24 years. Seven studies [35, 38–43] reported a mean age and two reported a median age [36, 37]. Two studies had narrow age ranges of up to 5 years, three studies had a broad age range of 20 years or more, while the remaining four studies did not report the age range. In one study, all mothers had a pre-pregnancy body mass index of 27 or above [37].

The strategies implemented in the studies included prenatal breastfeeding education (*n* = 2) [37, 42], peer support (*n* = 5) [35, 37–39, 42], professional support (*n* = 4) [36, 39, 40, 42], financial incentives (*n* = 1) [43], gift pack (*n* = 1) [41], telephone support (*n* = 5) [35, 36, 38, 39, 42] and massage (*n* = 1) [40]. Five of the studies included a peer counselling component and four of these used a combination of peer counselling and telephone support. Control groups received usual care or no treatment.

Four studies were included in the meta-analyses. Three of these were peer counselling interventions [35, 37, 39] and one involved education and telephone support [42]. The common follow-up time points across the included studies were 1-month, 3-months, and 6-months, however, only the 3-month time point was common across all four studies. The one-month time point was included in two studies [35, 37] and the six-month timepoint was included in three studies [37, 39, 42].

### Peer counselling

Peer counselling refers to support provided by a non-professional person from the community who has personal experience breastfeeding and a willingness to support others. Five studies included a peer counselling component [35, 37–39, 42]. Arlotti et al. [35] conducted a quasi-experimental study which included postnatal support from a peer counsellor a few days after birth, then at 2 weeks, 1 month, 2 months and 3 months postpartum. This support was delivered via telephone, letter or in person at the Women, Infants and Children (WIC) office.

In the RCT study by Chapman et al. [37], participants received three ‘specialized breastfeeding peer counselling’ sessions prenatally, daily visits in hospital after the birth, up to 11 postnatal sessions and routine care. Di Meglio et al. [38] conducted an RCT where the participants in the intervention group received telephone support from adolescent peer counsellors at 2, 4, and 7 days and 2, 3, 4, and 5 weeks after discharge from the hospital. The peer counsellors were trained by La Leche League and the content was based on WIC’s breastfeeding promotion materials.

In the RCT by Pugh et al. [39], the intervention group received usual care plus supplementary visits from a nurse and peer counsellor team. The visits were daily while the mothers were at the hospital, then at 1, 2, and 4 weeks postpartum when the mothers returned home. The peer counsellor provided telephone support twice weekly until 8 weeks postpartum followed by weekly telephone support until 6 months. Wambach et al. [42], included a lactation consultant and peer counsellor team who co-delivered two prenatal classes and provided telephone support over the first 4 weeks.

### Telephone support

Five studies included a telephone support component [35, 36, 38, 39, 42]. Only one study by Bunik et al. [36] used telephone support as its sole strategy and this support was delivered via a nurse (i.e., professional support). Four studies had a combination of telephone and peer counselling [35, 38, 39, 42] and two of these also included professional support via a lactation consultant or nurse [39, 42].

Bunik et al. [36] conducted an RCT where the mothers received 2 weeks of daily telephone calls from a bilingual nurse using a culturally informed script. Wambach et al. [42] conducted an RCT where the intervention group received a combination of prenatal classes and telephone support co-delivered by a lactation consultant and peer counsellor. The mothers also received in-hospital support from the peer counsellor and lactation consultant. Telephone support was provided at 4, 7, 11, 18 days, and 4 weeks.

### Prenatal education

Two studies included a prenatal education component [37, 42]. In the study by Chapman et al. [37], the education was delivered by peer counsellors who provided personalized breastfeeding education during three prenatal sessions. The peer counsellors were trained in La Leche League curricula [37]. In the study by Wambach et al. [42], a lactation consultant and peer counsellor co-delivered two prenatal classes based on Breastfeeding Educated and Supportive Teen club (BEST) curriculum. These studies also included a peer counselling element, with peer counselling provided in the home [37] or via telephone support [42].

### Other interventions

The remaining studies used a number of other strategies including massage [40], gift pack [41], and financial incentive [43]. Serrano et al. [40] conducted a quasi-experimental study where the participants were provided video instruction and a booklet on how to massage their baby. The aim was to evaluate the effect of massage on infant weight gain and EBF.

Snell et al. [41] conducted an RCT where mothers were randomly assigned to receive a gift pack which contained formula samples or not to receive a gift pack. As it is standard practice for USA hospitals to give formula samples to mothers, the “non-gift pack” group was the intervention group and the gift pack group was the control.

Washio et al. [43] conducted an RCT where the mothers received standard breastfeeding services from WIC plus monthly financial incentives, totaling USD $270 if they could demonstrate breastfeeding or pumping.

### Effectiveness of interventions on EBF

Overall there was modest to no effect with respect to increasing the EBF rates in the studies. Five studies reported no evidence of effect on rates of EBF [36, 37, 40, 42, 43]. These five studies included telephone support, peer counselling, massage, prenatal education, and financial incentives. Three studies reported a positive effect [35, 38, 41]. Two of these studies included a combination of peer counselling and telephone support in their intervention [35, 38]. Arlotti et al. [35], in their study involving peer counselling, reported a 36 percentage point difference in the mean rates of EBF to 2 weeks in the intervention versus usual care group (53% vs 17% respectively; *n* = 36). In the RCT by Di Meglio et al. [38], a statistically significantly longer duration of EBF was observed in the experimental group (telephone support from adolescent peer counsellors) compared to the usual care group (median 35 days vs 10 days, *p* = 0.01; *n* = 78). The study by Pugh et al. [39], which involved peer counselling, telephone support and a community health nurse, reported a non statistically significant positive effect of the intervention on rates of EBF (45% vs 25% to 3 months; *n* = 14; 30% vs 15% to 6 months; *n* = 9). Two studies, which used a combination of education and peer counselling [42] and financial incentives [43] found a statistically significant positive effect of the intervention for breastfeeding duration or breastfeeding rates but not for exclusive breastfeeding (Table 3).

**Table 3:** Effectiveness of interventions in included studies

Author, Year	N	Intervention	Results
Arlotti et al. (1998) [35]	36	Peer counselling (telephone, letter and in person at office)	Mean rates of EBF^, experimental VS control: • at 2 weeks, 53% vs 17% • at 1 month, 40% vs 27% • at 2 months, 33% vs 13% • at 3 months, 17% vs 6%
Bunik et al. (2010) [36]	341	Telephone support from a nurse	No mothers EBF
Chapman et al. (2013) [37]	206	Peer counselling including prenatal session (education), in-hospital and in-home postnatal sessions	EBF rates, experimental VS control: • at 1 month, 17.6% vs 12.1% (*p* = 0.37) • at 2 months, 11.9% vs 11.1% (*p* = 0.88) • at 3 months, 5.0% vs 9.4% (*p* = 0.49) • at 4 months, 1.6% vs 4.8% (*p* = 0.62) • at 5 months, 1.6% vs 1.6% (*p* = 0.999) • at 6 months, 1.7% vs 0.0% (*p* = 0.49)
Di Meglio et al. (2010) [38]	78	Peer counselling via telephone support	Duration of EBF, experimental vs control: Median 35 days vs 10 days, *P* < 0.01.
Pugh et al. (2002) [39]	41	Nurse/peer counselling team In-hospital, in-home and telephone support	Mean rates of EBF, experimental VS control: • at 3 months, 45% (*n* = 9) vs 25% (*n* = 5) • at 6 months, 30% (*n* = 6) vs 15% (*n* = 3) χ^2^ = 1.29–1.75; *P* = 0.09–0.12
Serano et al. (2010) [40]	100	Video instruction for baby massage delivered by nurses	Mean rates of EBF, experimental VS control: • at 2 months, 85.7% (*n* = 30) vs 81.54% (*n* = 53) • at 4 months, 71.4% (*n* = 25) vs 73.8% (*n* = 48) χ^2^ = 0.28–0.07; *P* = 0.595–0.795
Snell et al. (1992) [41]	88	Gift pack vs non gift pack	EBF rates, non-gift pack vs gift pack group: • at 1 week: 80% vs 68% (*p* > 0.05) • at 3 weeks: 68% vs 33% (*p* < 0.004)
Wambach et al. (2011) [42]	390	Lactation consultant and peer counsellor team Prenatal education, in-hospital and telephone support	EBF rates, experimental vs controls: • at 3 months: 32.14% vs 13.33% • at 6 months: 35.71% vs 22.22%
Washio et al. (2017) [43]	36	Financial incentives	No significant difference in self-reported EBF rate. (figures not reported)

### Quality assessment

**Fig. 2:**
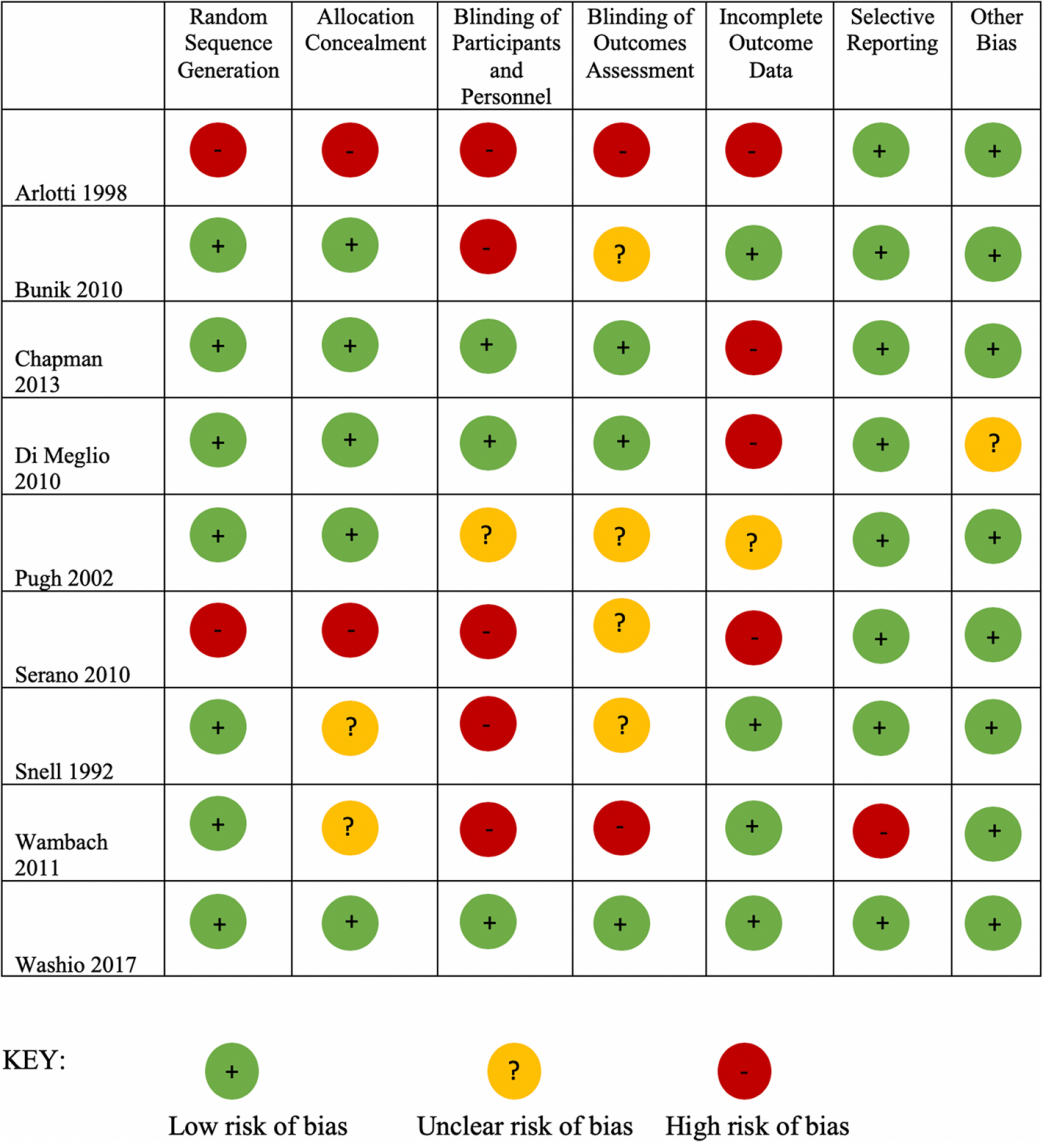
Quality assessment summary of studies included in the systematic review

The seven studies with an RCT design demonstrated a low risk of bias for random sequence generation [36–39, 41–43] and five of these were also deemed to have a low risk of bias for allocation concealment (Fig. 2) [36–39, 43]. The two studies with a quasi-experimental design did not randomize their participants and therefore, had a potential high risk of bias for both random sequence generation and allocation concealment [35, 40]. Blinding of participants, personnel and outcomes was limited for six of the nine studies, with either a high or unclear risk of bias [35, 36, 39–42]. Blinding was poorly described in the reports. Five studies were deemed to have high or unclear risk of bias in relation to incomplete data and attrition rates [35, 37–40]. Four studies had had low attrition rates and thus a low risk of bias in this domain [36, 41–43]. Most of the studies provided thorough reporting of outcomes and, given that the majority reported no effect, the risk of bias was deemed to be low in the selective reporting domain. One study [38] noted that Hispanic teens were less likely to participate in their study than Caucasian and African American teens, hence this was recorded as an ‘unclear’ risk of participation bias (in ‘other’).

### Meta-analyses

Between two and four studies were included in the meta-analyses, based on reporting of EBF outcomes, common time points and dichotomous data [35, 37, 39, 42]. The χ^2^ test and the I^2^ statistic for each analysis demonstrated sufficient homogeneity to combine the studies.

**Fig. 3:**
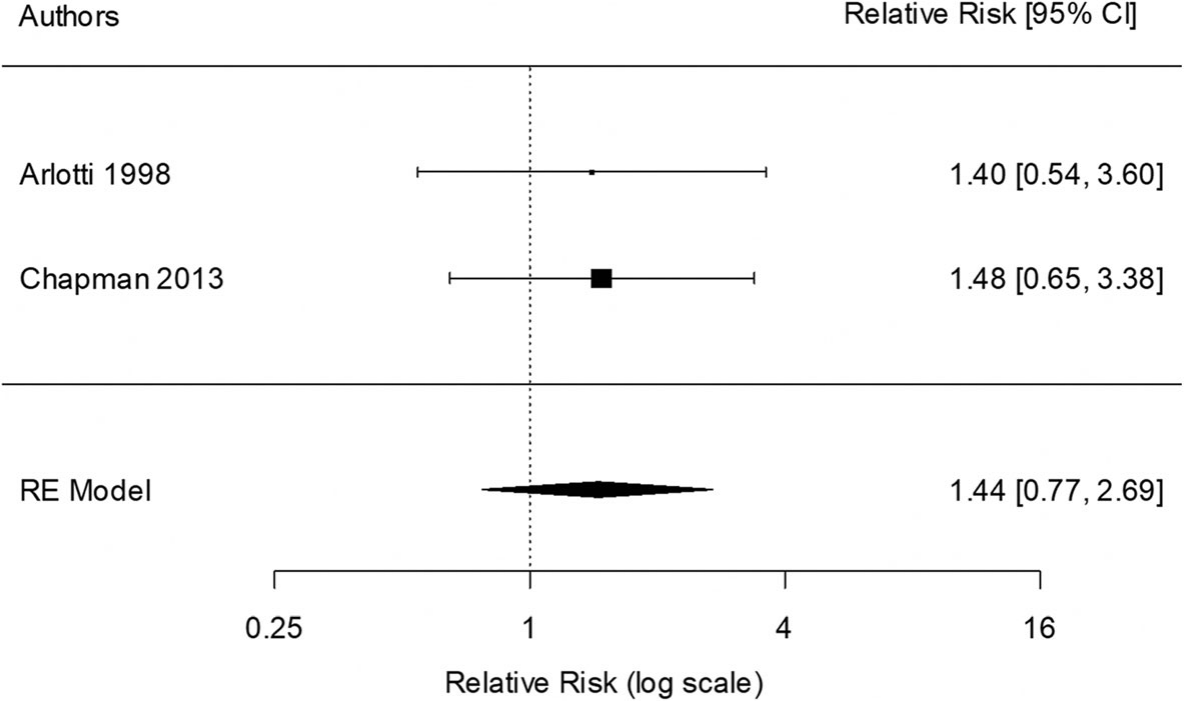
Meta-analysis of exclusive breastfeeding to 1 month

Two studies, both involving peer counselling, provided data for the 1-month analysis [35, 37]. There was no statistically significant effect of intervention on the rate of EBF to 1 month (RR 1.44; 95% CI 0.77, 2.69; *p* = 0.248; Fig. 3), compared with usual care. There was no evidence of heterogeneity (I^2^ = 0%; *p*-value 0.933). A sensitivity analysis for study quality was not possible due to the low number of included studies.

**Fig. 4:**
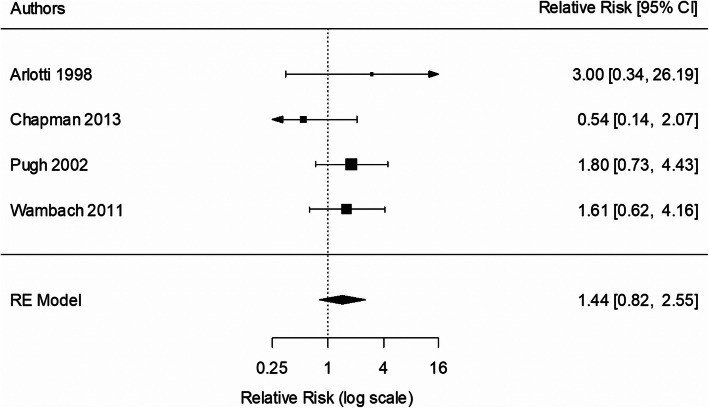
Meta-analysis of exclusive breastfeeding to 3 months

Four studies were included in the 3-month analysis [35, 37, 39, 42]. All four studies had a peer counselling element and three of the four studies [35, 39, 42] also included telephone support. Two studies had a prenatal education element [37, 42]. There was no statistically significant effect of intervention on the rate of EBF to 3 months (RR 1.44; 95% CI 0.82, 2.55; *p* = 0.204; Fig. 4), compared with usual care. There was no evidence of heterogeneity (I^2^ = 0%; *p* = 0.430). One study [35] was excluded (due to lower quality) for the sensitivity analysis. Sensitivity analysis indicated that the findings were unaffected by the study quality (RR 1.37, 95% CI 0.76, 2.46).

**Fig. 5:**
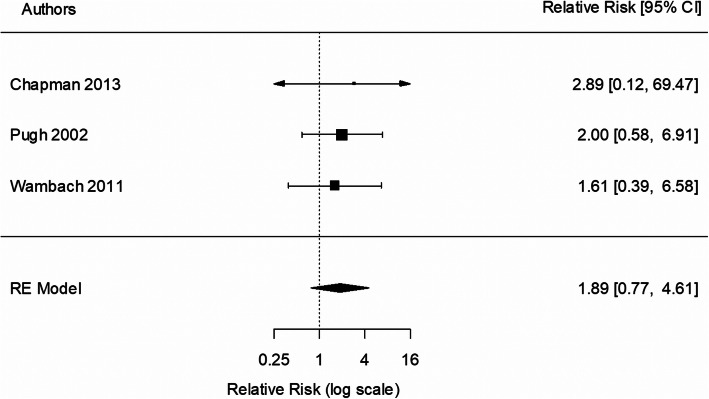
Meta-analysis of exclusive breastfeeding to 6 months

Three studies were included in the 6-month analysis [37, 39, 42]. All three studies included peer counselling, with two of the three studies [39, 42] including telephone support and two studies [37, 42] including prenatal education. There was no statistically significant effect of intervention on the rate of EBF to 6 months (RR 1.89; 95% CI 0.77,4.61; *p* = 0.164; Fig. 5), compared with usual care. There was no evidence of heterogeneity (I^2^ = 0%; *p* = 0.938). Exclusion of the lowest quality study from the analysis provided a RR of 2.10 (95% CI 0.66, 6.66).

## Discussion

This review sought to examine the range and effectiveness of interventions designed to increase rates of EBF among young mothers in high-income countries. Due to the heterogeneity of the interventions and the multiple-strategy nature of most of the interventions, it was not possible to conclude which strategies were most effective. However, interventions which involved peer counselling either as the main strategy or as one component of the overall intervention appear to be the most successful in increasing rates of EBF among young mothers. Two of the three studies which showed a significant positive effect on EBF used peer counselling as the principal strategy [35, 38]. A further study, Pugh et al. [39], which showed a non-statistically significant positive effect, also used peer counselling as the primary strategy.

There were however, several variations among the studies involving peer counselling. Peer counselling was delivered via different formats (telephone and in-person support) and at various time points across interventions, hence the optimal timing and format of peer counselling for this age-group of mothers is uncertain. For example, Chapman et al. [37] included three prenatal sessions and a large number of in-hospital and in-home postnatal sessions, whereas Arlotti et al. [35] delivered only five postnatal sessions. In two interventions, peer counselling was coupled with professional support from a nurse [39] or lactation consultant [42]. In the study by Di Meglio et al. [38] all peer counselling sessions were delivered by telephone whereas in the study by Arlotti et al. [35] only some sessions were delivered by telephone. The variety of formats used to deliver the peer counselling suggests that the exact format may not be important.

Several systematic reviews have examined the effectiveness of peer support in improving breastfeeding practices. Shakya et al. [49] and Jolly et al. [50] examined the effects across developed versus developing countries and indicated that this mode of intervention appears to be effective in developing countries but not developed countries. A review of 12 studies examining interventions designed to promote EBF in high-income countries by Skouteris et al. [51] showed that interventions with long-duration postnatal support components were effective at increasing EBF to 6 months.

There were two studies which combined prenatal education and peer support, and the education sessions were delivered by trained peer counsellors. In Chapman et al. [37] the peer counsellors delivered personalized one-on-one breastfeeding education, and in Wambach et al. [42] the peer counsellors facilitated two prenatal group classes. However, these two studies were ineffective at increasing the rate of EBF in young mothers in the USA [37, 42].

When compared to other breastfeeding education interventions, the systematic review by Lumbiganon et al. [24] also found no statistically significant evidence of effect of prenatal breastfeeding education on the rate of EBF among studies conducted predominantly in developed countries (RR 1.06 to 3 months; RR 1.07 to 6 months; pooled analyses for Summary of findings). However, Haroon et al. [52] demonstrated a positive effect of breastfeeding education on rates of EBF in both developed and developing countries, although with a stronger effect in those studies conducted in developing countries (RR 1.31 vs 2.88).

With respect to other strategies, only the ‘gift pack’ intervention conducted by Snell et al. [41] demonstrated a statistically significant effect on EBF. This may be relevant for countries in which hospitals still commonly practice giving gifts of formula to new mothers, however, may not be applicable to countries which have ceased this practice. The USA is one such country which does not adhere to the World Health Organization’s *International Code of Marketing of Breast-milk Substitutes* as infant formula advertising is widespread and free samples are often distributed in hospitals [53].

Eight of the nine included studies in the current review were conducted in the USA and as such, the findings may not be generalizable to other countries. Furthermore, many of the participants were from low socioeconomic communities or specific ethnic communities (such as Hispanic) and may not be generalizable to other communities. Although the rates of EBF in young mothers is low in most high-income countries, there are significant differences between high-income countries in relation to societal attitudes toward breastfeeding and system supports. For example, the USA only recently legalized breastfeeding in public in all 50 states (societal attitudes) and has no paid parental leave (system support) [53]. Thus, the factors and interventions influencing mothers from the USA could differ from the factors and interventions affecting mothers from other high-income countries.

The meta-analysis combined data from four of the nine included studies [35, 37, 39, 42]. Different outcome measurements made it difficult to compare studies, with varying follow-up times (2 weeks to 6 months) and various time points for data collection. Further, the data were sometimes expressed as continuous data (duration) and at other times dichotomous data (rates) so these were not able to be combined.

Overall, studies were of moderate quality. There was a lack of blinding and allocation concealment in many studies [35, 36, 39–42]. Sample attrition was common and randomization was not always possible or appropriate. It is possible that the results of interventions with large age ranges may not be reflective of what works with solely younger populations. The ages of the mothers who continued to exclusively breastfeed were not reported.

### Limitations

The findings of this systematic review and meta-analysis should be considered in light of several limitations. The review is limited by the relatively few studies which met the eligibility criteria. The sample sizes were small, with six of the nine studies having 100 or fewer participants. High rates of sample attrition and formula supplementation compounded this issue and made it difficult to detect a statistically significant effect. Because of the focus on high income countries in this review the results are not readily generalizable to low- and middle-income countries. Further, the included studies were mostly from the USA and may not be relevant to other high-income countries with different societal attitudes and system supports. As well, there was no exploration as to why mothers were not EBF or ceasing EBF early, as this was beyond the scope of the review.

As the eligibility for included studies was based on a mean or median age of less than 25 years, there were studies which had a wide age range. Without stratification it is not possible to determine whether the younger participants, specifically, found the intervention beneficial or not. This approach was necessary however as there were so few studies which included only mothers aged 24 years or younger and no studies were found which provided a sub-group analysis by age (possibly due to small numbers of younger mothers). The two studies [38, 42] which did, included only adolescent mothers (15–18 years) which was too narrow for the purpose of this review. Furthermore, the results of these two studies were not able to be combined in meta-analysis as the outcome measures were not compatible.

### Recommendations

More RCTs are required to test the effectiveness of interventions aimed at promoting rates of EBF among young mothers in high-income countries. Studies should specifically target young mothers (24 years or younger) or report the results of younger participants separately for studies conducted with a large age range. Young mothers have a unique set of characteristics, needs and barriers [28]. As such, it is important to understand the specific needs of young mothers so that interventions and health promotion programs can be tailored to suit them. It is recommended that future studies take into account blinding and allocation concealment to reduce potential for bias and increase reliability. Consistency in relation to follow up times would be advantageous so that intervention effectiveness can be more easily compared and/or findings combined for meta-analysis.

## Conclusions

Although this review included only a small number of studies, and the study populations differed in age range, there is an indication that peer counselling could be a promising intervention for improving rates of EBF among young mothers in high-income countries. This age group of mothers is understudied with respect to EBF promotion and support interventions. Intervention studies need to focus on young mothers or include sufficient numbers of young mothers to enable sub-group analysis by age.

## Supplementary Information

**Additional file 1:** **Appendix 1.** PRISMA Checklist.

**Additional file 2:** **Appendix 2.** Search Strategy example using the MEDLINE database.

**Additional file 3:** **Appendix 3.** Inclusion and exclusion criteria. Full list of inclusion and exclusion criteria.

**Additional file 4:** **Appendix 4.** List of excluded studies and reasons for exclusion.

**Additional file 5:** **Appendix 5.** Assessment of risk of bias in included studies – full table.

**Additional file 6:** **Appendix 6.** Raw data extracted for meta-analyses of exclusive breastfeeding to 1, 3, and 6 months.

## Data Availability

Not applicable.

## Abbreviations

BF: Breastfeeding
EBF: Exclusive breastfeeding
RCT: Randomisd controlled trial
RoBT: Risk of Bias Tool
WHO: World Health Organization
WIC: Women, Infants and Children office
