# Supplementary material for: Interventions to promote exclusive breastfeeding among young mothers: a systematic review and meta-analysis
Source: Int Breastfeed J. 2020 Dec 1;15:102. doi: 10.1186/s13006-020-00340-6 (PMC7706026; doi:10.1186/s13006-020-00340-6)
Supplement: Supplementary file 2 — Additional file 2: Appendix 2. Search Strategy example using the MEDLINE database. [file 13006_2020_340_MOESM2_ESM.docx]

**Additional file 2.** Search strategy example using MEDLINE database.

| **Searches** | **Results** |
| --- | --- |
| 1. exclusiv*.tw. | 164208 |
| 2. (breastfeeding or breast feeding or breastfed or breast fed).tw. | 43620 |
| 3. exp Breast Feeding/ | 37893 |
| 4. infant feeding.mp. | 5934 |
| 5. 2 or 3 or 4 | 58877 |
| 6. exp Adolescent/ or adolescent.mp. | 2066141 |
| 7. ("young mother"* or teen* or adolescen*).tw. | 926049 |
| 8. 6 or 7 | 2609361 |
| 9. 1 and 5 and 8 | 1722 |
| 10. exp Randomized Controlled Trial/ | 512876 |
| 11. random*.tw. | 1154724 |
| 12. randomized controlled trial.tw. | 76724 |
| 13. quasi-experimental.tw. | 13531 |
| 14. 10 or 11 or 12 or 13 | 1289801 |
| 15. 9 and 14 | 249 |
| 16. limit 15 to English language | 241 |
