# Supplementary material for: Interventions to promote exclusive breastfeeding among young mothers: a systematic review and meta-analysis
Source: Int Breastfeed J. 2020 Dec 1;15:102. doi: 10.1186/s13006-020-00340-6 (PMC7706026; doi:10.1186/s13006-020-00340-6)
Supplement: Supplementary file 3 — Additional file 3: Appendix 3. Inclusion and exclusion criteria. Full list of inclusion and exclusion criteria. [file 13006_2020_340_MOESM3_ESM.docx]

**Additional file 3.** Full list of inclusion and exclusion criteria.

|  | Inclusion Criteria | | Exclusion Criteria |
| --- | --- | --- | --- |
| - Written in English - Peer-reviewed - Human subjects - Measures exclusive breastfeeding - Mean or median age <25 - Conducted in high-income countries - RCT or quasi experimental design (prospective with control group) | | - Written in a language other than English - Not peer-reviewed - Animal subjects - Does not measure exclusive breastfeeding - Mean or median age 25+ - Conducted in low- or middle-income countries - Non RCT or quasi experimental study (retrospective or with no control group) | |
