# Supplementary material for: Interventions to promote exclusive breastfeeding among young mothers: a systematic review and meta-analysis
Source: Int Breastfeed J. 2020 Dec 1;15:102. doi: 10.1186/s13006-020-00340-6 (PMC7706026; doi:10.1186/s13006-020-00340-6)
Supplement: Supplementary file 4 — Additional file 4: Appendix 4. List of excluded studies and reasons for exclusion. [file 13006_2020_340_MOESM4_ESM.docx]

**Additional file 4.** List of excluded studies and reasons for exclusion.

| **Reason for exclusion** | **Excluded reference** | **Details** |
| --- | --- | --- |
| Not conducted in high income country  n=5 | Fewtrell 2011 | Belarus |
|  | Kupratakul 2010 | Thailand |
|  | Li 2014 | China |
|  | Sharma 2016 | India |
|  | Thukral 2012 | India |
| Study design  n=14 | Chalmers 2009 | This was a national survey. |
|  | Forster 2014 | Trial protocol. |
|  | Haroon 2013 | Systematic review |
|  | Hoddinott 2012(b) | Process evaluation |
|  | Jolly 2012 | Systematic review |
|  | Kronborg 2009 | Not an intervention (association) |
|  | Merten 2005 | Not an intervention (survey) |
|  | Nkonki 2017 | Systematic review |
|  | Perez-Escamilla | Systematic review |
|  | Relton 2016 | Study protocol |
|  | Renfrew 2012 | Systematic review |
|  | Shakya 2017 | Systematic review |
|  | Scott 2019 | Retrospective data analysis |
|  | Zakarija-Grkovic 2017 | Protocol |
| Mean/median age 25+ or unknown  n=28 | Ahmed 2016 | Mean age = 29 |
|  | Anderson 2005 | Mean age not reported. |
|  | Baerug 2016 | Mean age not reported. |
|  | Ball 2011 | Mean age = 31 |
|  | Bonuck 2014 | Mean age = 28 |
|  | Cattaneo 2001 | Mean age = 29 |
|  | Ching-Hsueh Yeh 2020 | Mean age = 31 |
|  | Dennis 2002 | Mean age not reported. |
|  | Ekstrom 2012 | Mean age = 26/27 |
|  | Elliott-Rudder 2014 | Mean age not reported. |
|  | Gallegos 2014 | Mean age = 31/30 |
|  | Gau 2004 | Mean age = 30 |
|  | Harari 2017 | Mean age = 26 |
|  | Hoddinott 2012(a) | Mean age = 29 |
|  | Ingram 2002 | Mean age = 29.5 |
|  | Jackson 2017 | Mean age = 29 |
|  | Jolly 2012 | Mean age not reported. 40% <25yr. |
|  | Laliberte 2016 | Mean age not reported. |
|  | Martinez-Brockman 2018 | Mean age = 26 |
|  | Nilsson 2017 | Mean age = 29 |
|  | Noel-Weiss 2006 | Mean age = 30 |
|  | Petrova 2009 | Mean age = 25 |
|  | Pollard 2011 | Mean age = 26/25 |
|  | Pound 2015 | Mean age not reported. |
|  | Pugh 1996 | Mean age = 26.4 |
|  | Relton, 2017 | Mean age = 37 |
|  | Sandy 2009 | Mean age = 26 |
|  | Srivinas 2015 | Age not reported at all. |
| Outcome  n=10 | Barbosa 2016 | Outcome was self-care. |
|  | Chapman 2013 | Acculturation and breastfeeding |
|  | Cloutier 2015 | Obesity in young children |
|  | Da Silva 2016 | Breastfeeding at 2 years |
|  | Edwards 2013 (complementary food) | Introduction of solids |
|  | Edwards 2013 (computer) | Intent to breastfeed exclusively |
|  | Hertzhaft-Le Roy 2017 | Outcome measure is latch. |
|  | Nicolson 2013 | Outcome measure is attachment. |
|  | Sussner 2009 | Older children aged 24-36 months. |
|  | Wilhelm 2015 | Intent to BF, self-efficacy, BF |
| Not in English n=1 | Lucchini 2013 | Spanish |
| Duplicate  n=2 | Bonuck 2013 | Duplicate of Bonuck 2014 |
|  | Yukkio, W. 2017 | Mislabelled duplicate – Washio. Y |
