# Supplementary material for: Interventions to promote exclusive breastfeeding among young mothers: a systematic review and meta-analysis
Source: Int Breastfeed J. 2020 Dec 1;15:102. doi: 10.1186/s13006-020-00340-6 (PMC7706026; doi:10.1186/s13006-020-00340-6)
Supplement: Supplementary file 5 — Additional file 5: Appendix 5. Assessment of risk of bias in included studies – full table. [file 13006_2020_340_MOESM5_ESM.docx]

**Additional file 5.** Assessment of risk of bias in included studies – full table.

Arlotti et al., 1998

| **Domain** | **Judgement** | | | **Quotes/Comments** |
| --- | --- | --- | --- | --- |
| **Selection Bias** |  |  |  |  |
| **Random sequence generation** | High | Low | Unclear | - Convenience sample - Not randomised. - To minimise confounds due to selection bias, data were collected on a number of motivational and social support variables. |
| **Allocation concealment** | High | Low | Unclear | - Women assigned based on their desire to have a counsellor and the availability of counsellors. Mothers not matched with a counsellor became part of the control group. |
| **Performance Bias** |  |  |  |  |
| **Blinding of participants and personnel** | High | Low | Unclear | - Participants and peer counsellors are not blinded. - It is unclear whether the participants and peer counsellors were aware of the nature of the study. |
| **Detection bias** |  |  |  |  |
| **Blinding of outcome assessment** | High | Low | Unclear | - For the intervention group, personal interviews (via telephone or in person) with the participant conducted by WIC nutritionist, peer counsellor, researcher or peer counsellor coordinator. For control group, contact was made by the researcher or WIC nutritionist. There is a high risk of bias as the same personnel involved in the delivery of the intervention are involved in the collection of the data. |
| **Attrition Bias** |  |  |  |  |
| **Incomplete outcome data** | High | Low | Unclear | - 10 participants lost to attrition. (36 participants completed the study). Those lost to attrition were statistically similar to the participants who completed the study except they were older (M=27.0 years old vs 23.3). - Does not provide information on why they dropped out or which group they were in. |
| **Reporting Bias** |  |  |  |  |
| **Selective reporting** | High | Low | Unclear | - Reporting appears thorough however no protocol is mentioned. - Primary and secondary outcomes appear to be reported. |
| **Other Bias** |  |  |  |  |
|  | High | Low | Unclear |  |

Bunik et al., 2010

| **Domain** | **Judgement** | | | **Quotes/Comments** |
| --- | --- | --- | --- | --- |
| **Selection Bias** |  |  |  |  |
| **Random sequence generation** | High | Low | Unclear | - Block random allocation |
| **Allocation concealment** | High | Low | Unclear | - Allocation assignment not blinded. - Sequentially numbered opaque sealed envelopes. |
| **Performance Bias** |  |  |  |  |
| **Blinding of participants and personnel** | High | Low | Unclear | - Not possible to blind participants and nurses conducting the intervention. |
| **Detection bias** |  |  |  |  |
| **Blinding of outcome assessment** | High | Low | Unclear | - Outcomes were assessed by maternal report over the telephone. - Unclear who conducted these interviews –was it the same bilingual nurses who conducted the intervention? - “We think we addressed bias in that only 1 of the 3 researchers who content coded the themes assisted in the interviews”. |
| **Attrition Bias** |  |  |  |  |
| **Incomplete outcome data** | High | Low | Unclear | - Low attrition 90% at 1 months follow up, 74% at 6 months |
| **Reporting Bias** |  |  |  |  |
| **Selective reporting** | High | Low | Unclear | - Thorough reporting and reported that the intervention was unsuccessful. |
| **Other Bias** |  |  |  |  |
|  | High | Low | Unclear |  |

Chapman et al., 2013

| **Domain** | **Judgement** | | | **Quotes/Comments** |
| --- | --- | --- | --- | --- |
| **Selection Bias** |  |  |  |  |
| **Random sequence generation** | High | Low | Unclear | - The study coordinator used SPSS software to randomly assign 50% of the participants to intervention group. |
| **Allocation concealment** | High | Low | Unclear | - The study coordinator used SPSS software to allocate 50% of the participants to intervention group, thus preserving allocation concealment. |
| **Performance Bias** |  |  |  |  |
| **Blinding of participants and personnel** | High | Low | Unclear | - Not possible to completely blind the participants however there was a similar support service available to the control group. - Peer Counsellors could not be blinded as they were conducting the intervention. |
| **Detection bias** |  |  |  |  |
| **Blinding of outcome assessment** | High | Low | Unclear | - Data collected by a different interviewer (not a peer counsellor). They were not informed of a participant’s group however the interviewer was not completely blinded as she collected PC contact data also. |
| **Attrition Bias** |  |  |  |  |
| **Incomplete outcome data** | High | Low | Unclear | - Intervention n=103; Control n=103 - High attrition (25/27 in each group) due to low birth weight, NICU, declined or BF contraindications. - Reasons for attrition similar in both groups. - Received allocated intervention n=76/78 (intervention/control) - Included in analysis n=55/53 - Lost around 30% to follow up. |
| **Reporting Bias** |  |  |  |  |
| **Selective reporting** | High | Low | Unclear | - Unlikely biased as they reported a negative outcome (no effect). - Reported a range of outcomes. |
| **Other Bias** |  |  |  |  |
|  | High | Low | Unclear |  |

Di Meglio et al., 2010

| **Domain** | **Judgement** | | | **Quotes/Comments** |
| --- | --- | --- | --- | --- |
| **Selection Bias** |  |  |  |  |
| **Random sequence generation** | High | Low | Unclear | - Principal Investigator (PI) used computer generated random numbers. |
| **Allocation concealment** | High | Low | Unclear | - Assignment recorded in a sealed number envelope. - Envelopes were sequentially opened as participants were recruited. |
| **Performance Bias** |  |  |  |  |
| **Blinding of participants and personnel** | High | Low | Unclear | - Only PI aware of group assignment and had no contact with participants. |
| **Detection bias** |  |  |  |  |
| **Blinding of outcome assessment** | High | Low | Unclear | - A single research assistant conducted all the telephone interviews using standardized, closed-ended questionnaires. The interviewer had no knowledge of the study hypothesis or design. - Patient reporting |
| **Attrition Bias** |  |  |  |  |
| **Incomplete outcome data** | High | Low | Unclear | - High drop out rate for peer counsellors – 3 of 5 excluded during first few assignments, 1 dropped out after 4 months, leaving only 1 peer counsellor involved throughout the entire study. - Intervention delivered inconsistently. |
| **Reporting Bias** |  |  |  |  |
| **Selective reporting** | High | Low | Unclear | - Thorough reporting. - Reported no effect. |
| **Other Bias** |  |  |  |  |
| **Monetary incentive** | High | Low | Unclear | - Participation incentive ($25 mall certificate) given to all participants who completed the 8 week interview regardless of BF outcomes. This minimised the possibility of participants altering their feeding decisions to obtain the monetary incentive. |
| **Participation bias** | High | Low | Unclear | - Hispanic teens more likely to decline to participate. Caucasian and African American more likely to participate. |

Pugh et al., 2002

| **Domain** | **Judgement** | | | **Quotes/Comments** |
| --- | --- | --- | --- | --- |
| **Selection Bias** |  |  |  |  |
| **Random sequence generation** | High | Low | Unclear | - Randomly assigned by sealed envelope technique to intervention or usual care group. (Does not specify how the random sequence was generated). |
| **Allocation concealment** | High | Low | Unclear | - Sealed envelope |
| **Performance Bias** |  |  |  |  |
| **Blinding of participants and personnel** | High | Low | Unclear | - Participants unable to be blinded. - Unclear if personnel are blinded. - The intervention and control groups receive similar support, but the intervention group receives supplementary visits. |
| **Detection bias** |  |  |  |  |
| **Blinding of outcome assessment** | High | Low | Unclear | - Does not specify who collects this data? Is it the same personnel delivering the intervention? |
| **Attrition Bias** |  |  |  |  |
| **Incomplete outcome data** | High | Low | Unclear | - Information not provided. |
| **Reporting Bias** |  |  |  |  |
| **Selective reporting** | High | Low | Unclear | - All outcomes discussed including non-statistically significant outcomes - BF duration, EBF, partial BF, and costs discussed. |
| **Other Bias** |  |  |  |  |
|  | High | Low | Unclear |  |

Serano et al., 2010

| **Domain** | **Judgement** | | | **Quotes/Comments** |
| --- | --- | --- | --- | --- |
| **Selection Bias** |  |  |  |  |
| **Random sequence generation** | High | Low | Unclear | - Quasi-experimental comparative panel design - Control = first visit occurred May-June 2004 - Intervention = first visit occurred July-September 2004 |
| **Allocation concealment** | High | Low | Unclear | - Not possible to conceal the allocation as the intervention was massage instruction. |
| **Performance Bias** |  |  |  |  |
| **Blinding of participants and personnel** | High | Low | Unclear | - Not possible for participants and nurses to be blinded |
| **Detection bias** |  |  |  |  |
| **Blinding of outcome assessment** | High | Low | Unclear | - Unclear whether the investigators were blinded but unlikely given that the intervention and control were conducted at 2 separate times. - It was good that the data was not collected by the investigators. |
| **Attrition Bias** |  |  |  |  |
| **Incomplete outcome data** | High | Low | Unclear | - High level of attrition - 65 control participants completed the visits (47.7%) - 35 massage participants completed the visits (20.59%) - There was a countrywide epidemic of acute upper respiratory tract infections. |
| **Reporting Bias** |  |  |  |  |
| **Selective reporting** | High | Low | Unclear | - Thorough reporting - Weaknesses and issues with data explored. - No significant difference reported. |
| **Other Bias** |  |  |  |  |
|  | High | Low | Unclear |  |

Snell et al., 1992

| **Domain** | **Judgement** | | | **Quotes/Comments** |
| --- | --- | --- | --- | --- |
| **Selection Bias** |  |  |  |  |
| **Random sequence generation** | High | Low | Unclear | - 12-week study period divided into two-week time blocks which were randomly assigned as either gift pack or non-gift pack periods. |
| **Allocation concealment** | High | Low | Unclear | - Not possible to conceal allocation (gift pack group received a gift pack and non-gift pack group did not receive a gift pack) |
| **Performance Bias** |  |  |  |  |
| **Blinding of participants and personnel** | High | Low | Unclear | - Not possible to blind participants and personnel (as above) |
| **Detection bias** |  |  |  |  |
| **Blinding of outcome assessment** | High | Low | Unclear | - It is unclear if the investigators were blinded |
| **Attrition Bias** |  |  |  |  |
| **Incomplete outcome data** | High | Low | Unclear | - Relatively few participants lost to follow up |
| **Reporting Bias** |  |  |  |  |
| **Selective reporting** | High | Low | Unclear | - Data is thoroughly reported - Demographic, obstetric and neonatal data gathered - Results at both time points reported (1 and 3 weeks) |
| **Other Bias** |  |  |  |  |
|  | High | Low | Unclear |  |

Wambach et al., 2011

| **Domain** | **Judgement** | | | **Quotes/Comments** |
| --- | --- | --- | --- | --- |
| **Selection Bias** |  |  |  |  |
| **Random sequence generation** | High | Low | Unclear | - Prospective, nonblinded, three-group RCT - Two control groups (usual care and attention control) - Randomly assigned using a list of random codes generated by the study biostatistician. |
| **Allocation concealment** | High | Low | Unclear | - Information not provided on this - Random codes generated by biostatistician |
| **Performance Bias** |  |  |  |  |
| **Blinding of participants and personnel** | High | Low | Unclear | - Nonblinded - But the attention control may have reduced bias as it mimicked the breastfeeding intervention to control for attention and other nonspecific effects. |
| **Detection bias** |  |  |  |  |
| **Blinding of outcome assessment** | High | Low | Unclear | - Nonblinded - No information on outcome blinding. |
| **Attrition Bias** |  |  |  |  |
| **Incomplete outcome data** | High | Low | Unclear | - Relatively low attrition rate - 390 enrolled. 315 (81%) provided baseline data; 289 (92%) of those provided analysable data. - Reasons for attrition clearly documented e.g. participant moved, pre-term labour, withdrew, non-adherence |
| **Reporting Bias** |  |  |  |  |
| **Selective reporting** | High | Low | Unclear | - Thorough reporting of statistical analysis. - Non-significant outcomes reported. - However, does not provide details of all outcomes e.g. EBF data collected until 6 months but only 3-week outcomes are mentioned. |
| **Other Bias** |  |  |  |  |
|  | High | Low | Unclear |  |

Washio et al., 2017

| **Domain** | **Judgement** | | | **Quotes/Comments** |
| --- | --- | --- | --- | --- |
| **Selection Bias** |  |  |  |  |
| **Random sequence generation** | High | Low | Unclear | - Randomized 2-arm parallel-group design. |
| **Allocation concealment** | High | Low | Unclear | - SAS PROC PLAN using allocation blocks of 2 were used by a statistician to ensure balance in the groups. |
| **Performance Bias** |  |  |  |  |
| **Blinding of participants and personnel** | High | Low | Unclear | - Participants and personnel were not blinded however the outcome is unlikely to be influenced by lack of blinding. |
| **Detection bias** |  |  |  |  |
| **Blinding of outcome assessment** | High | Low | Unclear | - Assessors were not blinded? - However, the outcomes were concrete, observable/tangible measurements |
| **Attrition Bias** |  |  |  |  |
| **Incomplete outcome data** | High | Low | Unclear | - All participants included in analysis (intent-to-treat analysis) - 1 loss to follow up in control group |
| **Reporting Bias** |  |  |  |  |
| **Selective reporting** | High | Low | Unclear | - Thorough reporting – primary and secondary outcomes reported; 3 time points; CONSORT diagram, inclusion/exclusion criteria clearly explained and justified. - Positive effect for BF outcome; No effect for EBF and infant outcomes. |
| **Other Bias** |  |  |  |  |
|  | High | Low | Unclear |  |
