# Supplementary material for: Interventions to promote exclusive breastfeeding among young mothers: a systematic review and meta-analysis
Source: Int Breastfeed J. 2020 Dec 1;15:102. doi: 10.1186/s13006-020-00340-6 (PMC7706026; doi:10.1186/s13006-020-00340-6)
Supplement: Supplementary file 6 — Additional file 6: Appendix 6. Raw data extracted for meta-analyses of exclusive breastfeeding to 1, 3, and 6 months. [file 13006_2020_340_MOESM6_ESM.docx]

**Additional file 6. Raw data extracted for meta-analyses of exclusive breastfeeding to 1, 3 and 6 months.**

1 month

| Name | n | n_T | success_T | failure_T | n_C | success_C | failure_C |
| --- | --- | --- | --- | --- | --- | --- | --- |
| Arlotti 1998 | 36 | 18 | 7 | 11 | 18 | 5 | 13 |
| Chapman 2013 | 133 | 67 | 12 | 55 | 66 | 8 | 58 |
| Wambach 2011 | 117 | 46 | 10 | 36 | 71 | 7 | 64 |

3 months

| Name | n | n_T | success_T | failure_T | n_C | success_C | failure_C |
| --- | --- | --- | --- | --- | --- | --- | --- |
| Arlotti 1998 | 36 | 18 | 3 | 15 | 18 | 1 | 17 |
| Chapman 2013 | 119 | 57 | 3 | 54 | 62 | 6 | 56 |
| Pugh 2002 | 40 | 20 | 9 | 11 | 20 | 5 | 15 |
| Wambach 2011 | 53 | 28 | 9 | 19 | 25 | 5 | 20 |

6 months

| Name | n | n_T | success_T | failure_T | n_C | success_C | failure_C |
| --- | --- | --- | --- | --- | --- | --- | --- |
| Chapman 2013 | 108 | 55 | 1 | 54 | 53 | 0 | 53 |
| Pugh 2002 | 40 | 20 | 6 | 14 | 20 | 3 | 17 |
| Wambach 2011 | 23 | 14 | 5 | 9 | 9 | 2 | 7 |

**Key:**

n = number of participants in study

n_T = number of participants in treatment group

success_T = number of participants exclusively breastfeeding (EBF) in treatment group

failure_T = number of participants not EBF in treatment group

n_C = number of participants in control group

success_C = number of participants exclusively EBF in control group

failure_C = number of participants not EBF in control group
